# Functional Conservation of P48/45 Proteins in the Transmission Stages of *Plasmodium vivax* (Human Malaria Parasite) and *P*. *berghei* (Murine Malaria Parasite)

**DOI:** 10.1128/mBio.01627-18

**Published:** 2018-09-04

**Authors:** Yi Cao, Robert J. Hart, Geetha P. Bansal, Nirbhay Kumar

**Affiliations:** a Department of Tropical Medicine, School of Public Health and Tropical Medicine, Tulane University, New Orleans, Louisiana, USA; b Vector-Borne Infectious Diseases Research Center, Tulane University, New Orleans, Louisiana, USA; Johns Hopkins Bloomberg School of Public Health

**Keywords:** malaria, P48/45, transgenic, transmission

## Abstract

Sexual-stage proteins have a distinct function in the mosquito vector during transmission and also represent targets for the development of malaria transmission-blocking vaccine. P48/45, a leading vaccine candidate, is critical for male gamete fertility and shows >50% similarity across various species of *Plasmodium*. We evaluated functional conservation of P48/45 in Plasmodium vivax and P. berghei with the motivation to establish transgenic P. berghei strains expressing P. vivax P48/45 (Pvs48/45) in an *in vivo* assay to evaluate the transmission-blocking activity of antibodies elicited by Pvs48/45. Homologous recombination was employed to target P. berghei *s48*/*45* (*pbs48*/*45*) for knockout (KO) or for its replacement by two different forms of P. vivax *s48*/*45* (*pvs48*/*45*) (the full-length gene and a chimeric gene consisting of *pbs48*/*45* 5′ signal and 3′ anchor sequences flanking *pvs48*/*45*). Expression of Pvs48/45 in transgenic parasites and lack of expression of any P48/45 in KO parasites were confirmed by reverse transcription-PCR (RT-PCR) and Western blotting. Both transgenic and knockout parasites revealed asexual growth kinetics in mice comparable to that seen with wild-type parasites. When employed in mosquito infection experiments, both transgenic parasite strains remained transmission competent and developed into infectious sporozoites, whereas the knockout parasites were incapable of establishing mosquito-stage infection. These results indicate the functional conservation of P48/45 protein during transmission, and the transgenic parasites generated in this study represent a valuable tool to evaluate the protective efficacy of transmission-blocking antibodies elicited by Pvs48/45-based vaccines using an *in vivo* mouse animal assay instead of *ex vivo* membrane feeding assays (MFA) relying on access to P. vivax gametocytes from infected patients.

## Abstract (precis)

Malaria transmission depends upon successful sexual differentiation and maturation of parasites in the vertebrate host and further development in the mosquito midgut. Stage-specific proteins in the sexual stages have been shown to play a critical role in development and successful transmission through the anopheline mosquito vector. Studies presented in the current manuscript evaluated functional conservation of one such protein, P48/45, in two diverse species (P. berghei and P. vivax). Replacement of endogenous *pbs48*/*45* in P. berghei with *pvs48*/*45* (P. vivax homologue) did not affect the viability of the parasites, and the transgenic parasites expressing Pvs48/45 remained transmission competent. These studies establish not only the functional conservation of P48/45 in P. berghei and P. vivax but also offer an opportunity to develop an *in vivo* test model for Pvs48/45-based P. vivax transmission-blocking vaccines, currently under development.

## INTRODUCTION

The transmission of *Plasmodium* parasites begins with commitment of asexually replicating blood-stage parasites to sexual-stage development, resulting in the formation of female and male gametocytes in the vertebrate host. Mature gametocytes are activated in the midguts of anopheline mosquitoes after a blood meal to produce gametes, followed by fertilization into zygotes. The zygote develops into the motile ookinete, which penetrates and traverses the midgut epithelium to form an oocyst under the basolateral lamina. Further sporogonic development involves production of sporozoites in the oocysts and migration of mature sporozoites to salivary glands for transmission to a vertebrate host during the next blood feeding process. These processes are obligatory for successful malaria transmission (1). Specific proteins in the various sexual stages of malaria parasites play important roles in these biological processes and are also antigenic targets for the development of malaria transmission-blocking vaccine (TBV). Antibodies against many of these target antigens when present in the blood meal effectively block parasite sexual development in the mosquito vector (2). The TBV target antigens are divided into two classes depending upon the time of their expression: (i) prefertilization antigens expressed in the gametocytes circulating in the vertebrate blood, such as P. falciparum 48/45 (Pfs48/45) (3) and Pfs230 (4) in P. falciparum and P. vivax 48/45 (Pvs48/45) (5) in P. vivax, and (ii) postfertilization antigens expressed on the surface of zygotes and ookinetes developing in the mosquito midguts, such as Pfs25 (6) in P. falciparum and Pvs25 and Pvs28 (7) in P. vivax.

Expression of *Plasmodium* P48/45 protein begins within developing intraerythrocytic gametocytes in the vertebrate host, and the expression persists on the surface of extracellular gametes in the mosquito midguts. These proteins belong to a six-cysteine protein family conserved in all *Plasmodium* species. The multiple conserved disulfide bonds in P48/45 proteins ensure the proper conformational folding that is critical for protein function, and such conformational epitopes represent key targets of transmission-blocking antibodies. Monoclonal antibodies against Pfs48/45 that effectively blocked transmission (8–10) provided the first direct evidence in support of the identification of P48/45 antigen as one of the leading TBV candidates. Further studies have validated P48/45 as an important target of transmission blocking immunity, including induction of potent transmission-blocking immune responses after immunization with recombinant Pfs48/45 (11–14) and Pvs48/45 (5) and DNA vaccines encoding Pfs48/45 (15) and Pvs48/45 (16). Antibodies against Pfs48/45 in human serum samples from areas of endemicity have revealed a strong correlation with natural transmission-reducing activity (17, 18). Large-scale genomic sequence analysis has revealed only limited rates of sequence polymorphism in *pfs48*/*45* (19) and *pvs48*/*45* (20, 21) compared to other erythrocytic and preerythrocytic stage-specific antigens being pursued as vaccine candidates.

Studies employing targeted gene disruption in P. falciparum and P. berghei have demonstrated a critical role for P48/45 in male gamete fertility (22), and the presence of P48/45 proteins is imperative for transmission of all *Plasmodium* species. While comparison of *p48*/*45* sequences among P. vivax, P. falciparum, and P. berghei has revealed 51% to 56% identity at the amino acid level and 60% to 67% identity at the DNA level (21), it is unknown whether functional domains important for sexual development are conserved across different species. We sought to address this issue via replacement of the *p48*/*45* gene in the rodent malaria parasite (P. berghei) by its homologue (*pvs48*/*45*) in the human malaria parasite (P. vivax) using homologous recombination. The choice to use *pvs48*/*45* for such gene replacement was largely dictated by the need to develop an animal model for evaluating P. vivax transmission-blocking vaccine based on Pvs48/45 using a rodent challenge model. An underlying hypothesis was that these studies would establish functional conservation of P48/45 in two different species of *Plasmodium* and also offer an opportunity for further development of a novel *in vivo* animal model to assess the protective efficacy of TBVs. Lack of an *in vitro* culture system for P. vivax further lends credence to the idea of the need to develop a convenient small-animal model. Currently, the only way in which transmission-blocking activity can be assessed is to conduct membrane feeding assays (MFAs) using blood from P. vivax-infected individuals (23).

In this study, transgenic P. berghei parasites were generated by genetically replacing endogenous *pbs48*/*45* with two different forms of *pvs48*/*45* (full-length and chimeric forms). The asexual-blood-stage growth of both transgenic parasites was comparable to that of wild-type (WT) parasites, and expression of Pvs48/45 in the transgenic parasites was demonstrated by reverse transcription-PCR (RT-PCR) and Western blot analysis. Our studies also revealed that both transgenic parasites were capable of undergoing complete sporogonic development in the anopheline mosquitoes, albeit at reduced levels compared to WT parasites. These results indicate the functional conservation of P48/45 protein in transmission of two diverse *Plasmodium* species, P. vivax and P. berghei. Efforts to develop a transmission-blocking vaccine based on Pvs48/45 are currently under way, and we believe that the transgenic parasites described in our studies will represent a valuable tool to evaluate the protective efficacy of transmission-blocking antibodies elicited by Pvs48/45-based vaccines using an *in vivo* mouse model instead of the tedious *ex vivo* MFAs relying on access to P. vivax gametocytes from infected patients.

## RESULTS

### Generation of transgenic P. berghei parasites.

**FIG 1:**
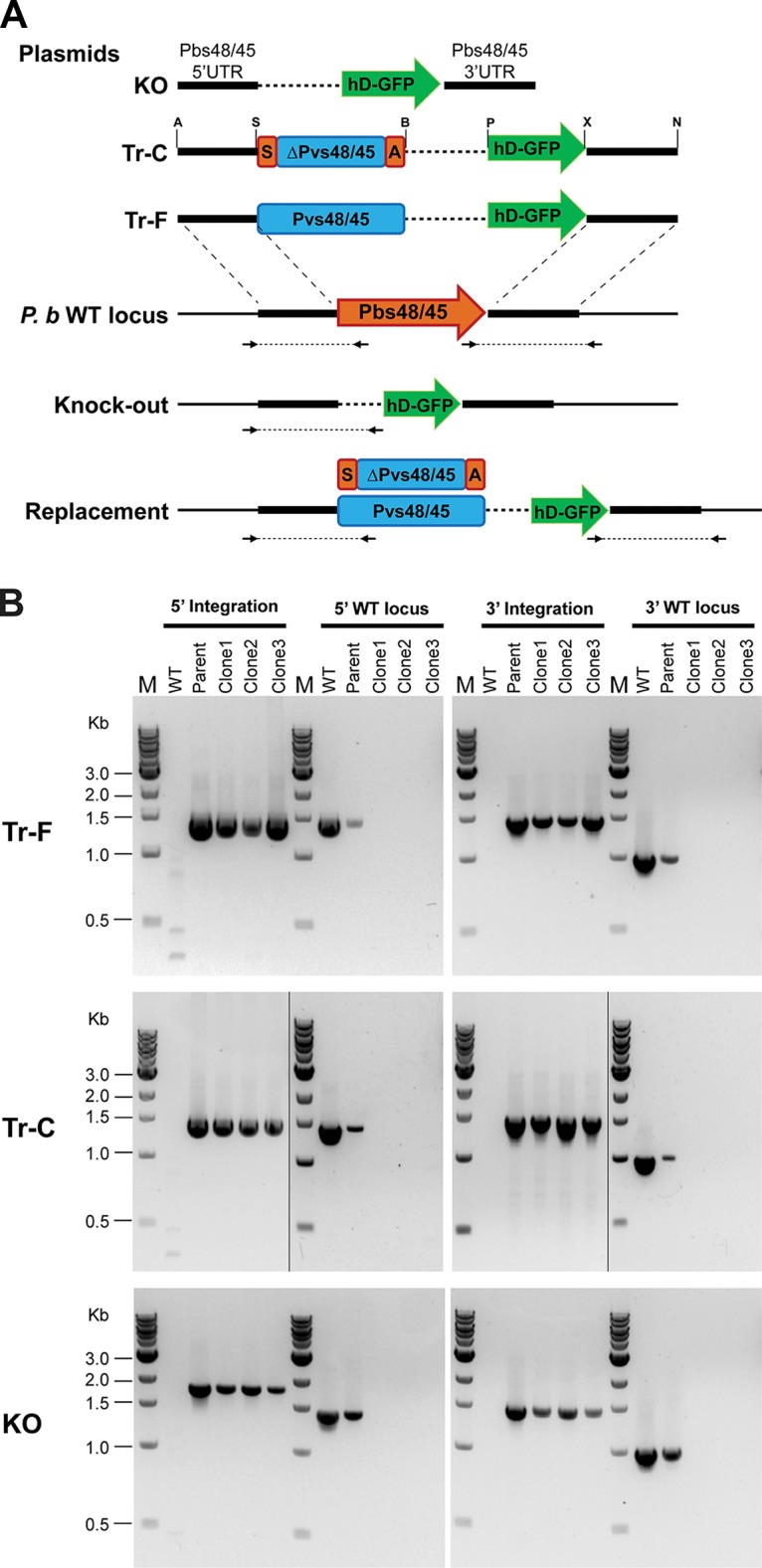
Schematic representation of gene-targeting plasmids and the resulting genomic loci after homologous recombination and integration. (A) The 5′ UTR and 3′ UTR sequences of *pbs48*/*45* gene were used to target the genomic locus in P. berghei (*P. b*) parasites by double-crossover recombination. *hd-gfp* represents the fused *hdhfr* and *gfp* genes for drug selection and fluorescent detection. The plasmid for KO contained *hd-gfp* flanked by 5′ and 3′ UTRs of *pbs48*/*45*. The plasmid used to generate Tr-F contained a cassette of full-length *pvs48*/*45* upstream of *hd-gfp* flanked by 5′ and 3′ UTRs of *pbs48*/*45*. The plasmid used to generate Tr-C contained a cassette of chimeric *pvs48*/*45* (internal *pvs48*/*45* gene sequence, ΔPv S48/45, ligated with signal and anchor regions of *pbs48*/*45*) upstream of *hd-gfp* flanked by 5′ and 3′ UTRs of *pbs48*/*45*. In the chimeric *pvs48*/*45*, signal and anchor regions of *pbs48*/*45* are indicated by boxes labeled “S” and “A,” respectively. Panel A also shows genomic loci after integration of KO, Tr-F, and Tr-C plasmids. The PCR primers used for confirmation of correct genomic integration and wild-type loci are shown by arrows. Restriction sites: ApaI (A), SacII (S), BglII (B), PstI (P), Xhol (X), and NotI (N). (B) PCR confirmation of correct genomic integrations at *pbs48*/*45* genomic locus in KO, Tr-C, and Tr-F parasites. The parent parasite population from each plasmid transfection contained both the wild-type and correctly integrated parasites. The 3 individual parasite clones with correct integration from each transfection plasmid were selected using specific PCR primers. The wild-type (WT) parasites were used as a control in PCR. Lanes marked M represent the DNA standards used to calibrate the gels.

The 5′ untranslated region (5′ UTR) and 3′ UTR sequences of the *pbs48*/*45* gene in the three targeting plasmids facilitated double-crossover recombination, resulting in parasites that lacked the native *pbs48*/*45* gene locus or parasites with native *pbs48*/*45* replaced by either a full-length or a chimeric *pvs48*/*45* gene. To generate the Pvs48/45-expressing transgenic parasite, the full-length *pvs48*/*45* sequence from P. vivax Sal-I strain was directly cloned into a targeting plasmid downstream of the *pbs48*/*45 5′ UTR* to replace the *pbs48*/*45* gene in P. berghei (Fig. 1A). We also designed a targeting plasmid with a chimeric sequence consisting of signal and anchor sequences from *pbs48*/*45* and the remainder sequence of *pvs48*/*45* (Fig. 1A). The rationale for the latter was that endogenous signal and anchor sequences might facilitate efficient trafficking of chimeric Pvs48/45 protein to the P. berghei parasite surface. After homologous recombination, the full-length and chimeric *pvs48*/*45* genes replaced the *pbs48*/*45* gene, but both were still under the control of the original *pbs48*/*45 5′ UTR* promoter region (Fig. 1A). As a control, the plasmid without any *pvs48*/*45* sequence was constructed to delete the entire *pbs48*/*45* gene, resulting in knockout (KO) parasites (Fig. 1A). After transfection and pyrimethamine drug selection, various transgenic parasites were characterized by diagnostic PCR for correct genomic integration (Fig. 1B). Three individual parasite clones from each of parent populations, confirmed for correct integration by diagnostic PCR using specific primers for both 5′ and 3′ integration, were selected (Fig. 1B).

### Expression of Pvs48/45 in transgenic P. berghei parasites.

**FIG 2:**
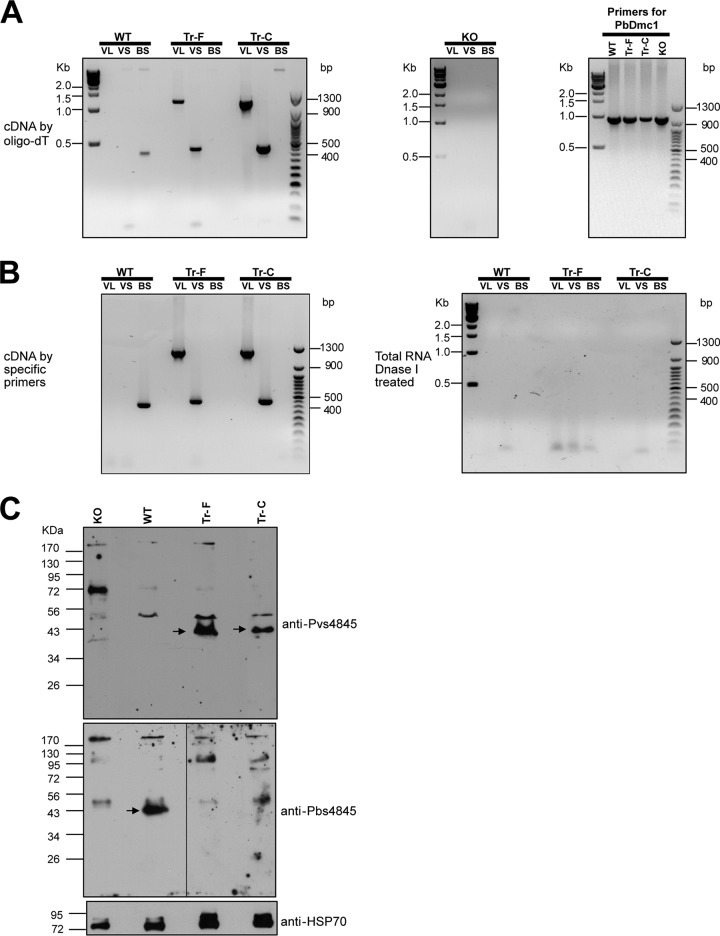
Transcription and expression of Pvs48/45 in transgenic parasites. (A) PCR detection using cDNAs prepared by reverse transcription using universal oligo(dT)18 primer. VL, primer pairs (VL-F and VL-R) for a long product (1,214 bp) from *pvs48*/*45*; VS, primer pairs (VS-F and VS-R) for a short product (452 bp) from *pvs48*/*45*; BS, primer pairs (BS-F and BS-R) for a short product (423 bp) from *pbs48*/*45* were used for PCR amplification. The PCR results from WT, Tr-F, and Tr-C parasites are shown in the left panel, while the results from KO parasite are shown in the middle panel. The quality of the cDNAs obtained from all the parasites was tested using primers for an unrelated gene (*pbdmc1*) as a positive control (right panel). (B) (Left panel) PCR detection using cDNAs prepared by reverse transcription using gene-specific primers. (Right panel) PCR results obtained using DNase I-treated RNA samples without reverse transcription. The use of the assay ruled out the possibility of genomic DNA contamination. (C) The gametocyte-enriched parasite proteins of WT, KO, Tr-F, and Tr-C were fractionated by SDS-PAGE, transferred to nitrocellulose membranes, and probed with anti-Pvs48/45 mouse serum samples (upper panel) and anti-Pbs48/45 rabbit serum samples (middle panel). Anti-PfHSP70 mouse serum samples were used as a control for confirmation of the quality of the parasite lysates (lower panel).

Transcripts of full-length and chimeric *pvs48*/*45* in Tr-F parasites (transgenic P. berghei expressing full-length Pvs48/45) and Tr-C parasites (transgenic P. berghei expressing chimeric Pvs48/45) as well as deletion of *pbs48*/*45* in the KO parasite were examined by RT-PCR using specific primers. Initially, all RNAs were reverse transcribed into cDNAs using universal oligo(dT)18 primer. Products with the sizes of 1,214 bp and 452 bp expected for *pvs48*/*45* transcripts were detected in the Tr-F and Tr-C parasites, respectively, but not in the WT parasites. In contrast, a 423-bp product corresponding to the *pbs48*/*45* transcript was detected only in WT parasites and not in the Tr-F parasites or the Tr-C parasites. The absence of any *pbs48*/*45* transcript in the KO parasite was confirmed by RT-PCR using the same set of primers (Fig. 2A). To further confirm the *pvs48*/*45* transcripts in the Tr-F and Tr-C parasites, the gene-specific primers were also used instead of oligo(dT)18 for reverse transcription of RNAs. Use of primer 889, consisting of sequence from the *pbs48*/*45* anchor region, resulted in the expected products in Tr-C and WT parasites, whereas use of primer 887, consisting of sequence from the *pvs48*/*45* anchor region, resulted in the expected products only in the Tr-F parasites (Fig. 2B). These results demonstrated that the full-length and chimeric *pvs48*/*45* genes were successfully transcribed in the Tr-F and Tr-C parasites, respectively.

We next looked for the expression of Pvs48/45 proteins in the transgenic parasites by Western blotting. The gametocyte-enriched extracts from WT, KO, Tr-F, and Tr-C parasites were fractionated by SDS-PAGE, transferred to nitrocellulose membranes, and probed with specific antisera. Anti-Pvs48/45 mouse serum samples recognized a protein band that was ~45 kDa in size in both Tr-F and Tr-C parasites but not in the WT parasites. When further probed with rabbit anti-P. berghei s48/45 (anti-Pbs48/45) antisera, a protein band of the expected size (~45 kDa) was recognized only in WT parasites and not in either Tr-F or Tr-C parasites. KO parasites failed to reveal any anti-Pvs48/45 or anti-Pbs48/45 reactive band of corresponding size (Fig. 2C). The quality of various parasite lysates was verified using anti-PfHSP70 antisera for probing the blots.

### Growth kinetics of Pvs48/45 transgenic and Pbs48/45 knockout parasites compared to wild-type parasites.

**FIG 3:**
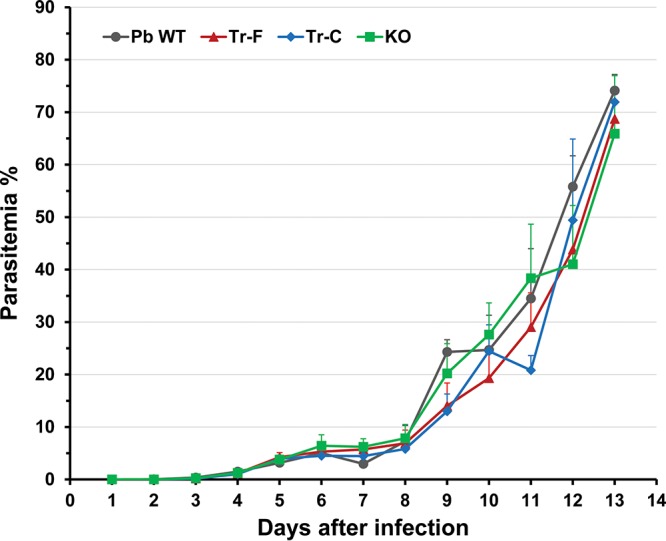
Blood-stage growth kinetics of WT, Tr-F, Tr-C, and KO parasites. Mice were infected with 2 × 10^5^ parasites intravenously (i.v.), and levels of parasitemia (percent infected erythrocytes) were measured daily by microscopic examination of Giemsa-stained blood smears. Data are shown as means ± standard deviations (SD) for 4 mice per group. Differences in growth kinetics were not statistically significant.

The blood-stage growth kinetics of Pvs48/45-expressing parasites (Tr-F and Tr-C), Pbs48/45 KO parasites, and WT parasites was investigated using mice infected with 2 × 10^5^ parasites and monitoring levels of parasitemia daily. As shown in Fig. 3, the blood-stage growth kinetics of both Pvs48/45 transgenic parasites and KO parasite were comparable to WT parasite kinetics. The replacement or knockout of endogenous *pbs48*/*45* did not affect the blood-stage growth kinetics of the P. berghei parasite in the vertebrate host. We also determined levels of gametocytemia in mice infected with Tr-F, Tr-C, KO, and WT parasites on the days typically used for mosquito transmission studies (i.e., on day 4, 5, or 6 after initiation of blood-stage infection). As shown in Fig. S2 in the supplemental material, the Tr-F, Tr-C, KO, and WT parasites had comparable levels of gametocytemia on all 3 days.

### Transmission competence of Pvs48/45 transgenic and Pbs48/45 knockout parasites compared to wild-type parasites.

**FIG 4:**
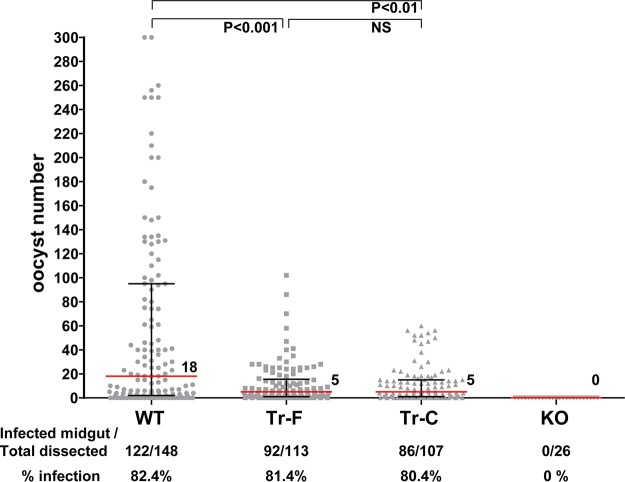
Comparison of levels of transmission competence of WT, Tr-F, Tr-C, and KO parasites in A. stephensi mosquitoes. Oocyst numbers of mosquitoes fed on 8 infected mice (4 experiments with 2 mice per experiment) were pooled for each of the groups of WT, Tr-F, and Tr-C parasites. KO parasites were evaluated in only a single mosquito feeding experiment (2 mice). Horizontal red lines represent medians of oocyst numbers. The data corresponding to the rates of infection represent percent mosquitoes infected (infected mosquitoes/total number of mosquitoes dissected) and are shown at the bottom. Median oocyst numbers of WT parasites were significantly higher than those of Tr-F and Tr-C parasites. No statistically significant difference (NS) was detected between Tr-F and Tr-C parasites (*P* values are indicated and were determined by the Kruskal-Wallis test followed by Dunn’s test for specific group pairs). The Fisher’s exact test was used to compare the rates of infection and indicated that the differences between the results determined for WT, Tr-F, and Tr-C parasites were not significant. Note that 7 points of WT data are outside the *y*-axis limit.

The transmission competence of Pvs48/45 transgenic parasites was compared to that of WT parasites using Anopheles stephensi mosquitoes for transmission. In some limited experiments, we also tested the transmission competence of Pbs48/45 knockout parasites. The rates of infectivity (percent infected mosquitoes) were comparable between WT parasites and both the Pvs48/45 transgenic parasites (82.4% for WT versus 81.4% for Tr-F and 80.4% for Tr-C). However, the infectivity (oocyst number per midgut) of WT parasites (median, 18) was significantly higher than that of Tr-F parasites (median, 5; *P* = 0.0005) and Tr-C parasites (median, 5; *P* = 0.001) (Fig. 4). The total numbers of mosquitoes fed on mice infected with WT, Tr-F, and Tr-C parasites in several repeat experiments and then dissected were 148, 113, and 107, respectively. In contrast, Pbs48/45 KO parasites failed to infect A. stephensi mosquitoes. In addition, two different clones for each of groups of Pvs48/45 transgenic parasites (Tr-F and Tr-C) were initially tested and were found to be comparable with respect to their transmission competence (Fig. S3). The comparable levels of gametocytemia in the blood of mice infected with Tr-F, Tr-C, KO, and WT parasites ruled out the possibility that the reduced infectivity of Tr-F and Tr-C parasites with respect to mosquito vector was due to reduced gametocyte production in Tr-F and Tr-C parasites compared to WT parasites (Fig. S2). These findings demonstrate that transgenic P. berghei parasites expressing heterologous Pvs48/45 protein remain transmission competent and also suggest that Pvs48/45 protein is able to partially complement the function of endogenous Pbs48/45 protein during sexual development and transmission of parasites from the vertebrate host to the mosquito vector.

**FIG 5:**
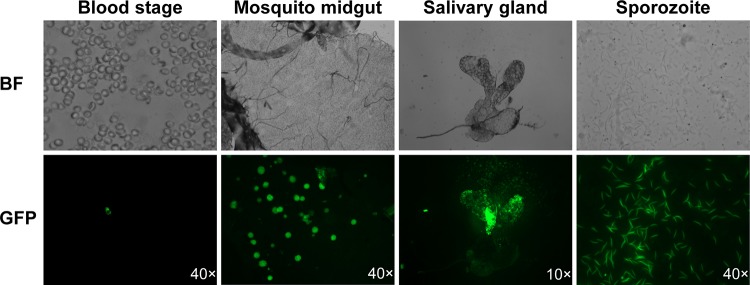
GFP expression in blood-stage and mosquito-stage transgenic parasites. The transgenic parasite clones with full-length or chimeric *pvs48*/*45* sequences displayed GFP signal in the blood-stage parasites, midgut oocysts, and salivary gland sporozoites. The fluorescence in this figure represents Tr-F parasites. BF, bright field images.

To follow various developmental stages of parasites in the vertebrate host and mosquito vector, we made use of green fluorescent protein (GFP) expression in the transgenic parasites. The transfection plasmids used to produce transgenic parasites contained a *gfp* gene fused in frame with the human dihydrofolate reductase gene (*hdhfr*) used as a drug-selectable marker. The expression of hDHFR was under the control of the *pbef 5′ UTR* promoter, constitutively active in all the life cycle stages of parasites. All the transgenic parasites with full-length or chimeric *pvs48*/*45* sequences displayed GFP expression in the blood-stage parasites, midgut oocysts, and salivary gland sporozoites (Fig. 5). Although both versions of the Pvs48/45 transgenic P. berghei parasites produced fewer midgut oocysts than the WT parasites, both supported further development of sporozoites and accumulation in the mosquito salivary glands (Fig. 5). The infectivity of sporozoites produced by transgenic parasites was confirmed by allowing these mosquitoes to feed on mice. Infection in mice in the Giemsa-stained blood smears was verified as well as development of blood-stage parasites expressing GFP (not shown).

## DISCUSSION

During malaria transmission, parasites growing as replicative erythrocytic asexual stages must first differentiate and mature into infectious gametocytes which, upon ingestion by the mosquito, initiate a cascade of developmental steps. These include gametogenesis (formation of extracellular male and female gametes), fertilization of gametes, and transformation of zygote into ookinete. Motile ookinetes traverse the midgut wall and develop into oocysts wherein sporozoites are produced which then migrate to the salivary gland for further transmission. During these sexual developmental life cycle steps, a number of proteins are expressed which are presumed to play important roles in this development process. P48/45 protein investigated in this study has been shown to play a critical role in the fertilization process during sexual development. In a previous study, targeted disruption of P48/45 genes in P. falciparum and P. berghei was found to severely impair formation of zygote and ookinete and fertility in mosquito feeding experiments. Other studies have shown that antibodies against P48/45 effectively interfere with the fertilization process, resulting in significantly reduced oocyst burden and thus providing the rationale for the development of a malaria transmission-blocking vaccine based on P48/45 antigen. While those studies clearly pointed out the importance of P48/45 proteins, it is not known if they can mutually complement biological function in heterologous species of *Plasmodium*. Sequences of *p48*/*45* in various *Plasmodium* species share greater than 50% identity at both the gene and protein sequence levels, and nearly all transmission-blocking antibodies in different species of *Plasmodium* have been shown to be directed against conformational epitopes formed by pairing of highly conserved cysteine residues.

We wished to first investigate functional conservation of P48/45 in different species of *Plasmodium*. Our approach involved development of transgenic P. berghei (rodent malaria parasites) expressing Pvs48/45 of P. vivax instead of endogenous Pbs48/45. The choice of *pvs48*/*45* as a transgene was based on the fact that there is no culture system to propagate P. vivax in culture, and we rationalized that functional conservation would pave the way for further development of an *in vivo* small-animal model system to evaluate P. vivax transmission-blocking vaccine. Transgenic P. berghei expressing Pvs48/45 protein in the sexual stages and in an appropriate conformation may serve as a tool to test the effect of Pvs48/45-specific antibodies after vaccination during transmission from mice to mosquitoes. We used full-length *pvs48*/*45* as well as a chimeric *pvs48*/*45* gene (consisting of signal and anchor sequences of *pbs48*/*45* and the remaining internal sequence of *pvs48*/*45*) to generate transgenic parasites. The rationale for the latter was that the homologous signal and anchor sequences might function more efficiently than the sequences from a heterologous species in P. berghei parasites. However, we did not find any difference in protein expression results between the transgenic parasites expressing full-length Pvs48/45 or chimeric Pvs48/45 in P. berghei. Our studies also confirmed that the expression of P48/45 protein is not critical for the development of intraerythrocytic asexual and sexual stages. The transgenic parasites (Tr-F and Tr-C) and the knockout parasites showed comparable asexual growth kinetics results in mice. Lack of expression of P48/45 in the knockout parasites and expression of Pvs48/45 instead of Pbs48/45 in the transgenic parasites were confirmed by Western blotting using specific antisera. Previous studies performed with Pbs48/45 knockout P. berghei parasites had provided similar results, i.e., comparable asexual and sexual development data in mice (22).

Next, we evaluated whether transgenic parasites were transmission competent and capable of establishing infection in the mosquitoes. The transmission competence results were compared among Pvs48/45-transgenic parasites, KO parasites, and WT parasites on the basis of oocyst production in the mosquito midguts. The comparisons of oocyst production levels between two Pvs48/45-transgenic parasite strains with full-length or chimeric Pvs48/45 did not reveal any statistically significant difference. Oocyst production in both Pvs48/45-transgenic parasite strains was lower than in WT parasites, whereas the KO parasites were completely incompetent. P48/45 protein expression begins in the intraerythrocytic gametocytes in the vertebrate circulation, and the expression persists on the surface of extracellular gametes and zygotes, stages found in the mosquito midguts. Previous studies based on targeted gene disruption had suggested that it is critical for male gamete fertility. Biochemical studies had also shown that P48/45 physically interacts with P230 (another surface protein in the sexual stages) and forms a stable membrane-bound complex. Elucidation of the biological significance of such interaction in gamete fertilization remains elusive. It has been speculated that P48/45 may function via interactions with other parasite proteins on the surface of gametes. Since transgenic P. berghei parasites expressing both full-length and chimeric Pvs48/45 were transmission competent, a similar experimental approach based on replacement of select domains might facilitate further identification of functional domains. It is possible that the replacement of endogenous Pbs48/45 in P. berghei with Pvs48/45 might weaken but not eliminate the molecular interactions between P48/45 and other proteins in P. berghei, resulting in reduced efficiency of gamete fertilization. However, we do not have any experimental evidence to support such speculation.

Our results obtained with knockout parasites are somewhat different from previous findings obtained using Pbs48/45 knockout parasites (22). In those studies, knockout of Pbs48/45 in P. berghei significantly impaired but did not completely eliminate gamete fertilization or ookinete and oocyst formation, and the authors speculated that their results might indicate partially impaired development due to an unknown alternative fertilization pathway. The most significant observations revealed by the results determined in the current study are that Pvs48/45 was able to complement the function of Pbs48/45, at least in part, during P. berghei gamete fertilization and ookinete formation and that the Pvs48/45-transgenic parasites were transmission competent in the mosquito vector. Furthermore, sporozoites of GFP-expressing transgenic parasites were observed in the salivary glands of mosquitoes and were infective with respect to naive mice by mosquito bite or intravenous injection of isolated sporozoites.

The evaluation of transmission-blocking activity for human malaria parasites critically depends on a robust biological assay known as the membrane feeding assay. Among the various parameters (24), the standard membrane feeding assay (SMFA) is considered a “gold standard” for analysis of P. falciparum. It involves feeding a mixture of culture-derived mature P. falciparum gametocytes and test antibodies to *Anopheles* mosquitoes through a membrane feeding apparatus and measuring subsequent mosquito infection. This assay has been utilized broadly in both preclinical and clinical studies. In the case of P. vivax, the lack of an *in vitro* culture method has hampered the development of a similar standard assay to assess activity of P. vivax TBV candidates. The *ex vivo* membrane feeding assay used in limited published studies has employed P. vivax gametocytes in freshly collected blood from infected patients (23). The use of different sources of gametocytes from infected patients introduces assay-to-assay variability, making any serious analysis difficult. Studies have also attempted to develop surrogate assays to measure malaria transmission of P. falciparum or P. berghei by examining gametocyte, gamete, or ookinete production *in vitro* (24). For example, assays based on examining P. berghei ookinete formation (25) or P. falciparum gametocyte viability (26, 27) have been employed to screen malaria transmission-blocking drugs and vaccines in a high-throughput manner. However, all these assays again require culture methods that can serve as a source of infectious gametocytes, which is not currently feasible for P. vivax.

The functional conservation of P48/45 during malaria transmission and the fact that the transgenic parasites developed in this study were transmission competent now open the possibility of deploying them to assess transmission-blocking antibodies against Pvs48/45.

The use of SMFA or mosquito feeding assays with transgenic Tr-F or Tr-C parasites is one way to use them to evaluate the transmission-blocking activity of specific antibodies. We are currently in the process of developing and characterizing a panel of Pvs48/45 monoclonal antibodies. When available, it will be possible to assess their functional activity using transgenic parasites in SMFA. We have also recently succeeded in expressing recombinant Pvs48/45 in Escherichia coli, and we are trying to optimize the conditions for protein refolding to achieve the correct epitope conformations that are critical to elicit the transmission-blocking antibodies. We have also developed DNA vaccine plasmids, and the DNA plasmids were found in initial immunization studies to be weaker immunogens. Availability of monoclonal antibodies and/or polyclonal antisera will allow us to test transgenic parasites in future studies.

Alternatively, these transgenic parasites can also be employed in a passive transfer approach using an *in vivo* mouse model. Such an assay would employ infection of mice with these Pvs48/45 transgenic P. berghei parasites and would assess transmission to mosquitoes in direct feeding assays after passive transfer of previously generated antibodies. Similar approaches have been developed for a few other malarial antigens. For example, transgenic P. berghei parasites expressing P25 of P. falciparum (28) and P. vivax (29), another leading TBV candidate, were developed as the animal malaria model alternatives to the *ex vivo* mosquito membrane feeding assays (MFA) to evaluate transmission-blocking activity of the serum samples against P25 from nonhuman primate (28) and human (29) clinical trials.

## MATERIALS AND METHODS

### Ethical statement.

All the research on mice adhered to the “Principles of Laboratory Animal Care” (NIH publication number 85-23, revised in 1985), and the research was reviewed and approved by the Institutional Animal Care and Use Committee of Tulane University (approval protocol number 4172R2). Animals were monitored weekly for any adverse effects, and none of the animal became ill or died. At the end of the study period, animals were anesthetized with isoflurane for terminal blood collection by cardiac puncture.

### Construction of plasmids to knock out the endogenous *pbs48*/*45* gene or to replace it with full-length or chimeric *pvs48*/*45*.

The P48/45 proteins contain an amino-terminal signal sequence and a carboxy-terminal anchor sequence, important for proper subcellular trafficking of protein to the surface of extracellular gametes of *Plasmodium*. The *p48*/*45* genes of P. vivax and P. berghei share 60% and 51% identity in their DNA and amino acid sequences, respectively (21). Plasmids were designed to either knock out the endogenous *pbs48*/*45* gene (KO) or replace it with a *pvs48*/*45* gene to obtain transgenic P. berghei expressing full-length Pvs48/45 protein in place of endogenous Pbs48/45 (Tr-F). The levels of amino acid identity between Pbs48/45 and Pvs48/45 in the signal and anchor sequences were only 11% and 37%. Another plasmid was also designed to obtain transgenic parasites expressing chimeric *pvs48*/*45* sequence consisting of *pbs48*/*45* signal and anchor regions flanking the *pvs48*/*45* sequence, presumably for efficient subcellular trafficking (Tr-C). The coding sequence for green fluorescent protein (GFP) was included in all transfection plasmids to aid in the characterization of transfected parasites.

Plasmid pL0006 (MRA-775; BEI Resources, Inc.) was modified by replacing *pbef 5′ UTR-hdhfr* with a cassette consisting of *pbef 5′ UTR-hdhfr-gfp*. Using pL0006 as the template, *pbef 5′ UTR* and *hdhfr* regions were amplified using primers 858/859 and 860/861, respectively (Table 1). PCR-amplified *pbef 5′ UTR* was cloned by TA cloning in pCR2.1-TOPO vector (Invitrogen), followed by insertion of PCR-amplified *hdhfr* into the MluI and SalI sites. Next, the PCR-amplified *gfp* gene (amplified using primers 862/863 from plasmid pL0021 [MRA-790; BEI Resources, Inc.]) was cloned into pCR2.1-TOPO vector downstream of *hdhfr* using SalI and BamHI sites. Forward primer 862, used for amplification, also contained 5 codons to insert a linker of five alanine residues (shown in italics and underlined in Table 1) to separate hDHFR and GFP polypeptide domains within the fusion protein. The gene cassette containing *pbef 5′ UTR* and a fused *hdhfr-gfp* gene in the pCR2.1-TOPO vector was used to replace the fragment of *pbef 5′ UTR-hdhfr* in pL0006 using PstI and BamHI to obtain plasmid pL0006-*gfp* (see details in Fig. S1 in the supplemental material).

10.1128/mBio.01627-18.1 FIG S1  Schematic representation of generation of the pL0006-*gfp* plasmid. (A) Plasmid pCR2.1-TOPO was used to assemble *pbef 5′ UTR*, *hdhfr*, and *gfp* to generate an intermediate plasmid. The region between the T7 promoter (T7) and M13 reverse priming site (M13) of pCR2.1-TOPO, containing a T/A cloning site (T) and multiple cloning sites (MCS), is shown in the upper panel. When *pbef 5′ UTR* was cloned into pCR2.1-TOPO by T/A cloning, forward primer 858 for *pbef 5′ UTR* was designed to introduce the PstI restriction site into pCR2.1-TOPO, and reverse primer 859 was designed to introduce MluI and SalI restriction sites (middle panel). Next, *hdhfr* was inserted into pCR2.1-TOPO using MluI and SalI restriction sites, followed by insertion of *gfp* using SalI and BamHI restriction sites (lower panel). The BamHI site is original in the MCS of pCR2.1-TOPO downstream of T/A cloning site. (B) Plasmid pL0006 (upper panel) was digested with PstI and BamHI to delete both *pbef 5′ UTR* and *hdhfr*, which were replaced with the gene cassette containing *pbef 5′ UTR-hdhfr-gfp* obtained by PstI and BamHI digestion of the intermediate plasmid (lower panel in panel A), resulting in the plasmid pL0006-*gfp* (lower panel in panel B). Restriction sites used were as follows: ApaI (A), BamHI (Ba), BglII (B), EcoRI (E), KpnI (K), MluI (M), PstI (P), SacII (S), SalI (Sa), SpeI (Sp), Xhol (X), NotI (N). Download FIG S1, TIF file, 1.4 MB. Copyright © 2018 Cao et al. 2018 Cao et al. This content is distributed under the terms of the Creative Commons Attribution 4.0 International license.

10.1128/mBio.01627-18.2 FIG S2  Blood-stage levels of gametocytemia in mice infected with WT, Tr-F, Tr-C, and KO parasites. Levels of gametocytemia were measured in the same mice as those described in the Fig. 3 legend on days 4, 5, and 6 postinfection by microscopic examination of Giemsa-stained blood smears. Data are shown as means ± SD for 4 mice per group, and the differences were not statistically significant. Download FIG S2, TIF file, 1.1 MB. Copyright © 2018 Cao et al. 2018 Cao et al. This content is distributed under the terms of the Creative Commons Attribution 4.0 International license.

10.1128/mBio.01627-18.3 FIG S3  Comparable levels of transmission competence of the two different clones of Tr-F and Tr-C parasites in A. stephensi mosquitoes. Oocyst numbers of mosquitoes for the Tr-F clone 1 and Tr-C clone 1 represent the same data as the “Tr-F” and “Tr-C” data in Fig. 4, respectively. An independent clone of Tr-F and Tr-C parasites (designated clone 2) was evaluated in the mosquito feeding experiments on 4 infected mice for each parasite (2 experiments with 2 mice per experiment). No statistically significant difference (NS) was detected in the medians of oocyst numbers between the 2 clones of Tr-F and Tr-C (Kruskal-Wallis test followed by Dunn’s test for specific group pairs). The Fisher’s exact test was used to compare the rates of infection and also indicated no significant difference between the 2 clones of Tr-F and Tr-C. Download FIG S3, TIF file, 1.6 MB. Copyright © 2018 Cao et al. 2018 Cao et al. This content is distributed under the terms of the Creative Commons Attribution 4.0 International license.

**TABLE 1:** Pairs of primers^a^

Primer name	Gene region	Primer sequence	Template	Size (bp)
858	*pbef* 5′ UTR	F (5′-GATctgcagCCCAGCTTAATTCTTTTCAAGCTCTTTATGCTTA-3′)–**PstI**	Plasmid pL0006 (MRA-755)	605
859		R (5′-GATgtcgacacgcgtCCCTATGTTTTATAAAATT-3′)–**SalI-MluI**		
				
860	hDHFR	F (5′-GATacgcgtATGGTTGGTTCGCTAAAC-3′)–**MluI**	Plasmid pL0006 (MRA-755)	579
861		R (5′-GGAgtcgacATCATTCTTCTCATATACTTC-3′)–**SalI**		
				
862	GFP	F (5′-CGgtcgac*GCAGCAGCAGCAGCA*GGATCTAGTAAAGGAGAAGAAC-3′)–**SalI**^b^	Plasmid pHDGFP	751
863		R (5′-CGggatccTTATTTGTATAGTTCATCC-3′)–**BamHI**		
				
864	Pbs48/45 5′ UTR	F (5′-GTgggcccTTTAGAAATATTAACAGGGAG-3′)–**ApaI**	P. berghei WT gDNA	1,018
865		R (5′-GTccgcggTATTAAAGAGAGAAAAGGGACAC-3′)–**SacII**		
				
866	Pvs48/45 ORF	F (5′-GTccgcggATGTTGAAGCGCCAGCTCGCCAA-3′)–**SacII**	P. vivax Sal-1 strain gDNA	1,369
887		R (5′-GTagatctTCAGAAGTACAACAGGAGGAGCAC-3′) &–**BglII**		
				
870	Pbs48/45 3′ UTR	F (5′-GTctcgagAGTAGTGTGTAGCGTATTCTTTTATTTTAC-3′)–**XhoI**	P. berghei WT gDNA	582
886		R (5′-AAGAAgcggccgcGTATTCGAATTCAGTATTGCACGACTA-3′)–**NotI**		
				
884	P. berghei DHFR-TS 3′ UTR	F (5′-GTagatctCGTTTTTCTTACTTAT-3′)–**BglII**	Plasmid pL0006 (MRA-755)	570
885		R (5′-GTctgcagCATCGAAATTGAAGGAA-3′)*–**PstI**		
				
872	Trunc-Pvs48/45 ORF	F (5′-GAGGTCAAGTACGTCCCGCCAGAG-3′)	P. vivax Sal-1 strain gDNA	1,230
873		R (5′-CTTGGCGAGGAAGCCAAAGTAGC-3′)		
				
874	Pbs48/45 signal	F (5′-GTccgcggATGCTCTACTTTTTTGGGAACAG-3′) %–**SacII**	P. berghei WT gDNA	113
892		R (5′-GACGTACTTGACCTCACCAACTGACGATTTTATAACTAATAC-3′)		
				
874	Chimeric Pvs48/45 ORF	F (5′-GTccgcggATGCTCTACTTTTTTGGGAACAG-3′) %–**SacII**	Trunc-Pvs48/45 ORF, Pbs48/45 signal and anchor	1,378
889		R (5′-GTagatctTTATAGCCACATAAAAAATAAGG-3′) ◇–**BglII**		
				
896	5′ Integ (full and chimeric)	F (5′-CGTATGGGTATGAATGCACAACAATGG-3′)@	gDNAs of WT, Tr-F, Tr-C	1,437 (full length), 1,446 (chimeric)
897		R (5′-GCTCCTCTGGCGGGACGTACTTGACC-3′)		
				
896	5′ Integ (KO)	F (5′-CGTATGGGTATGAATGCACAACAATGG-3′)@	gDNAs of WT, KO	1,802
885		R (5′-GTctgcagCATCGAAATTGAAGGAA-3′)*		
				
899	3′ Integ	F (5′-GCGATGGCCCTGTCCTTTTACCAGAC-3′)	gDNAs of WT, Tr-F, Tr-C, KO	1,481
900		R (5′-GGCGTGCCGAAATTAAATAAAATCCTCTC-3′)$		
				
896	5′ Pbs48/45	F (5′-CGTATGGGTATGAATGCACAACAATGG-3′)@	gDNAs of WT, Tr-F, Tr-C, KO	1,440
898		R (5′-ATTCATCTGGAGAAACATACTCATTT-3′)		
				
901	3′ Pbs48/45	F (5′-GTTGGAGCAATACCTCAATCAGCATC-3′)	gDNAs of WT, Tr-F, Tr-C, KO	1,010
900		R (5′-GGCGTGCCGAAATTAAATAAAATCCTCTC-3′)$		
				
887	Reverse-transcribed RNA of full-length Pvs48/45	R (5′-GTagatctTCAGAAGTACAACAGGAGGAGCAC-3′) &	Total RNA of Tr-F	
				
889	Reverse-transcribed RNAs of Pbs48/45 and chimeric Pvs48/45	R (5′-GTagatctTTATAGCCACATAAAAAATAAGG-3′) ◇	Total RNA of WT and Tr-C	
				
908	RT-PCR Pvs48/45 (L)	F (5′-CGCCAGAGGAGCTGAACAAAGACG-3′)	cDNAs of WT, Tr-F, Tr-C, KO	1,214
909		R (5′-CTTGGCGAGGAAGCCAAAGTAGC-3′)		
				
910	RT-PCR Pvs48/45 (S)	F (5′-CGTTGACAGCACGATTTACACTTTGT-3′)	cDNAs of WT, Tr-F, Tr-C, KO	452
911		R (5′-CGGCGGCAATTTTAACGGAC-3′)		
912	RT-PCR Pbs48/45	F (5′-GTATTAAAATATCCCCATAAAATAGTATCTG-3′)	cDNAs of WT, Tr-F, Tr-C, KO	423
913		R (5′-TAAATTCATATTATTAGTAAATGTGCGAA-3′)		
				
658	RT-PCR Pbdmc1	F (5′-ATCGATGATGTGCGAAGAACCATTTGC-3′)	cDNAs of WT, Tr-F, Tr-C, KO	945
849		F (5′-CTGTTGCTTTCAGATAGCTCGC-3′)		

a The RE sites used for cloning are indicated with lowercase characters. Restriction enzymes are indicated in bold. The symbols *, %, @, $, &, and ◇ are used to indicate identical primers. The identical primers are listed repeatedly for instances where they were used again to make a pair of PCR primers for a given amplification reaction. F, forward; R, reverse; WT, wild-type P. berghei; Tr-F, transgenic P. berghei expressing full-length Pvs48/45; Tr-C, transgenic P. berghei expressing chimeric Pvs48/45; TS, thymidylate synthase; Trunc, truncated; Integ, integration; L, long; S, short; KO, Pbs48/45 knockout P. berghei; ORF, open reading frame; gDNA, genomic DNA.

b Five codons used to insert a linker of five alanine residues are indicated with italics and underlining.

To generate the knockout (KO) plasmid for homologous recombination by double crossover, *pbs48*/*45 5′ UTR* and *pbs48*/*45 3′ UTR* fragments were obtained by PCR amplification using genomic DNA as the template and primers 864/865 and 870/886, respectively. The *pbs48*/*45 5′ UTR* fragment was inserted into plasmid pL0006-*gfp* using ApaI and SacII sites (Fig. S1). Likewise, the *pbs48*/*45 3′ UTR* fragment was inserted in the Xhol and NotI sites. In addition, the *pbdhfr-ts 3′ UTR* (*pbdt 3′ UTR*) gene was amplified from plasmid pL0006 using primers 884/885 and inserted between the BglII and PstI restriction sites to obtain the KO plasmid for *pbs48*/*45* knockout in P. berghei parasites. The complete sequence of *pvs48*/*45* from P. vivax Sal-I genomic DNA extracted from dried blood spots on filter paper (BEI Resources, Inc.) was amplified using primers 866/887 and a QIAamp DNA Blood Minikit (Qiagen) and was inserted between the SacII and BglII restriction sites in the KO plasmid. The resulting plasmid was used to obtain transgenic P. berghei expressing full-length Pvs48/45 (Tr-F) instead of endogenous Pbs48/45. To generate the chimeric sequence of *pvs48*/*45* flanked by *pbs48*/*45* signal and anchor sequences, the truncated *pvs48*/*45* fragment without N-terminal signal and C-terminal anchor sequences was amplified using primers 872/874. The 110-bp signal sequence of *pbs48*/*45* was created by PCR using primers 874/892, and the resulting sequence contained 15 bp overlapping the 5′ end of the truncated *pvs48*/*45* fragment. A synthetic sequence (60 bp) representing the *pbs48*/*45* anchor region (5′ ttatagccacataaaaaataagggaataaatataataaacaaCTTGGCGAGGAAGCCAAA 3′) was synthesized by Genescript, Inc. (NJ, USA). The synthetic anchor sequence contained an 18-bp overlap (uppercase in the sequence) with the 3′ end of truncated *pvs48*/*45* fragment. The truncated *pvs48*/*45* fragment, secretory signal sequence, and anchor sequence were all used as the templates in PCR amplification using primers 874/889 to obtain the chimeric *pvs48*/*45* sequence containing the internal *pvs48*/*45* sequence flanked by the *pbs48*/*45* signal and anchor sequences, and the PCR fragment was inserted into the KO plasmid using SacII and BglII restriction sites to generate the plasmid used to produce transgenic P. berghei expressing chimeric Pvs48/45 (Tr-C).

### Parasite transfection, cloning, and fluorescence detection.

Plasmid DNAs were linearized by digestion with ApaI and NotI before parasite transfection. The P. berghei parasites were transfected using 10 µg linearized plasmid DNA and selected by treatment of the infected mice using pyrimethamine mixed in drinking water as described previously (30). The correct integration of plasmid DNA into the P. berghei genome was confirmed by PCR analysis using specific primer pairs (Fig. 1A; see also Table 1). The 5′ integration of plasmids used for replacement of the endogenous *pbs48*/*45* gene with either full-length or chimeric *pvs48*/*45* was verified using primers 896/897. Primers 896/885 were used to verify 5′ integration of the plasmid used to knock out *pbs48*/*45*. The 3′ integration of all the plasmids was verified by using primers 899/900. The 5′ UTR and 3′ UTR of wild-type *pbs48*/*45* genomic loci were verified using primers 896/898 and 901/900, respectively. Three parasite clones with correct integration for each of transgenic and KO parasites were obtained by the limiting dilution method. Since the plasmids were designed to integrate GFP coding sequence upon transfection, various cloned parasites were examined by the use of a fluorescence microscope (Olympus BX41) equipped with a QIClic charge-coupled-device (CCD) camera (QImaging), and captured images were processed by the use of QCapture Pro 7 software (QImaging). Live parasites were collected from tail blood of infected mice, and midguts and salivary glands of infected mosquitoes were examined to investigate expression of GFP in various vertebrate and mosquito stages of the parasites.

### RNA extraction and reverse transcription-PCR (RT-PCR).

The parasites were harvested from blood collected from Swiss-Webster mice infected with WT, KO, and transgenic P. berghei parasites. White blood cells were removed using cellulose columns. Total RNA was extracted from the parasite pellets by the use of Trizol reagent (Invitrogen) and dissolved in RNase-free pure water, and concentrations were determined using a NanoDrop spectrophotometer (model 2000/2000c). Two micrograms of total RNA was treated with DNase I (Invitrogen) per the product manual to deplete the reaction mixtures of genomic DNA contamination. A 1-µg volume of DNase I-treated RNA was reverse transcribed into cDNA using oligo(dT)18 primer and a RevertAid first-strand cDNA synthesis kit (Thermo Fisher) under the cycling conditions specified in the product manual. In addition, total RNA was also reverse transcribed using the following gene-specific primers: primer 889 for *pbs48*/*45* (WT) and chimeric *pvs48*/*45* (Tr-C) and primer 887 for full-length *pvs48*/*45* (Tr-F). cDNAs were used to investigate expression of full-length or chimeric Pvs48/45 in the transgenic parasites and also to demonstrate the lack of expression of Pbs48/45 in the KO parasite. Two different *pvs48*/*45* gene-specific primer pairs, 908/909 and 910/911, were used to amplify 1,214-bp-long and 452-bp-long regions of *pvs48*/*45* transcripts, respectively, while primer pair 912/913 was used to detect a 423-bp product corresponding to wild-type *pbs48*/*45* transcripts and primer pair 658/849 was used to demonstrate uninterrupted transcription of an unrelated gene, *pbDmc1*, as a positive control. The PCR cycling conditions were as follows: initial denaturation at 94°C for 1 min followed by 30 cycles of denaturation at 94°C for 20 s, annealing at 55°C for 30 s, and extension at 68°C for 1.5 min. All the RNA samples after DNase I treatment were tested without reverse transcription in parallel to rule out the possibility of amplification from contaminating genomic DNA instead of from the RNA template.

### Western blotting.

The gametocyte-enriched parasites were collected from infected Swiss-Webster mice by sulfadiazine treatment as described previously (31). The parasites were released from red blood cells by 0.1% saponin lysis for 10 min on ice and washed twice with ice-cold phosphate-buffered saline (PBS). The parasites were resuspended in the lysis buffer (50 mM Tris-HCl, 10 mM EDTA, 1 mM phenylmethylsulfonyl fluoride [PMSF], 1% Triton X-100, 1× protease inhibitor cocktail [Sigma]) and kept on ice for 1 h to extract the total proteins from parasite pellets. Lysed parasites were centrifuged, and proteins in the supernatants were fractionated using 12.5% SDS-PAGE gels under reducing conditions and transferred to nitrocellulose membranes. Membranes were probed with anti-Pbs48/45 rabbit serum (a kind gift from Melissa R. van Dijk, Leiden University, Netherlands) or anti-Pvs48/45 mouse serum (32) at the indicated dilutions and developed using horseradish peroxidase (HRP)-conjugated anti-rabbit or anti-mouse IgG antibody (1:10,000 dilution) and ECL Prime Western blotting detection reagent (GE Healthcare).

### Comparison of blood-stage parasite growth kinetics and transmission to mosquitoes.

The transgenic parasites with full-length and chimeric *pvs48*/*45* as well as *pbs48*/*45*-knockout parasites were compared to WT P. berghei parasites for their asexual growth kinetics. BALB/c mice (female, 6 to 8 weeks old) were infected intravenously with 2 × 10^5^ WT, KO, or transgenic parasites (4 mice/group). Levels of parasitemia were monitored daily by examining Giemsa-stained thin blood smears. The data were expressed as means ± standard deviations (SD), and statistical differences were analyzed using the Mann-Whitney test.

The transmission competence of transgenic, KO, and WT parasites was evaluated in A. stephensi mosquitoes. Swiss-Webster mice were infected with 10^6^ parasites through the intraperitoneal route, and on day 5 postinfection, starved A. stephensi female mosquitoes were fed on the infected mice, which had been anesthetized with ketamine and xylazine. Blood-fed mosquitoes were maintained in incubators at 19°C with 80% relative humidity using 10% dextrose-dipped cotton balls. Mosquito midguts were dissected 10 days after feeding to enumerate oocysts after staining with 0.1% mercurochrome. The data were analyzed statistically using the nonparametric Kruskal-Wallis test, followed by Dunn’s test for specific group pairs.
