# Supplementary figures and images for: Functional Conservation of P48/45 Proteins in the Transmission Stages of Plasmodium vivax (Human Malaria Parasite) and P. berghei (Murine Malaria Parasite)
Source: mBio. 2018 Sep 4;9(5):e01627-18. doi: 10.1128/mBio.01627-18 (PMC6123445; doi:10.1128/mBio.01627-18)

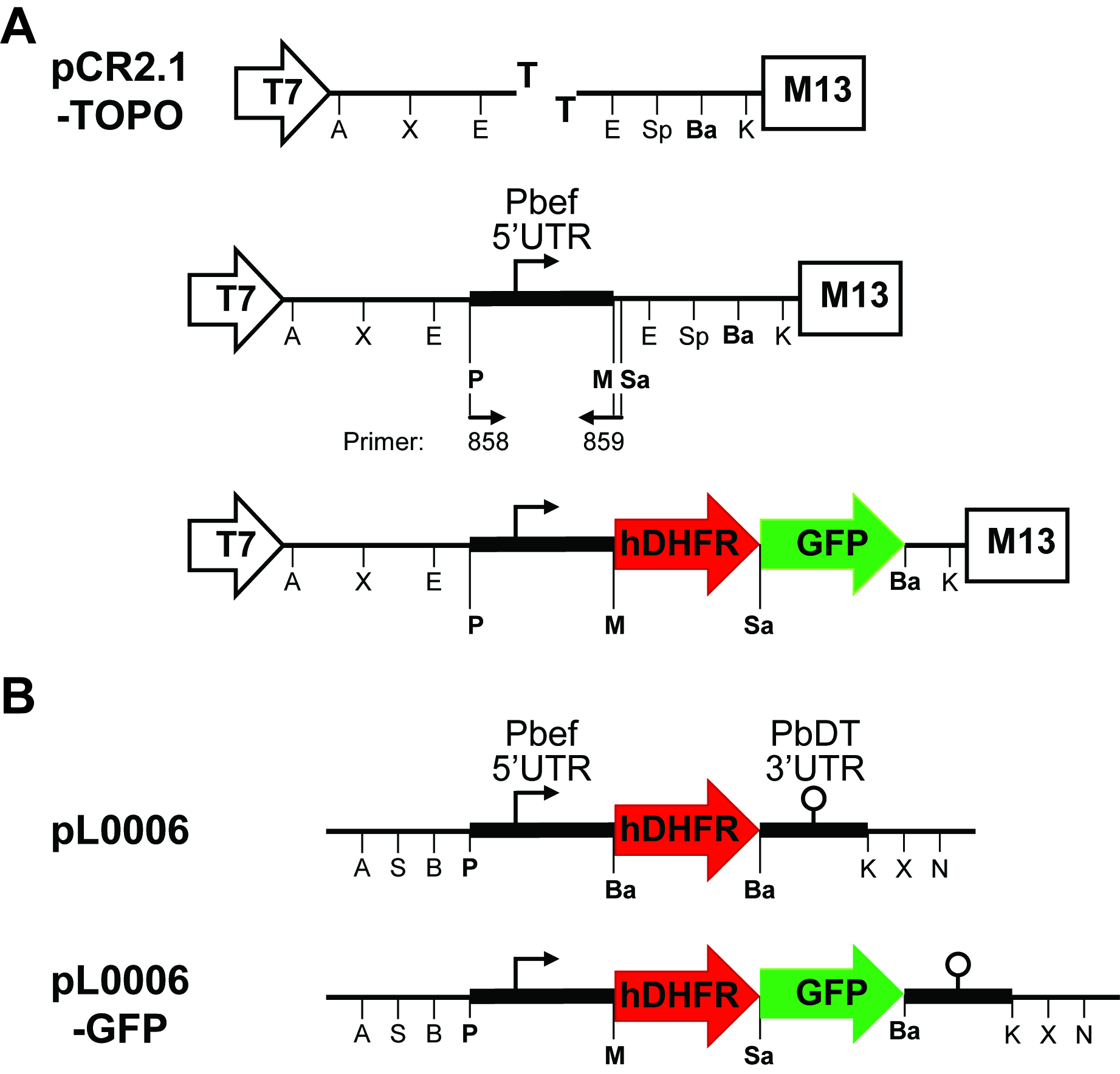

Supplement: FIG S1 [file mbo004184050sf1.tif]

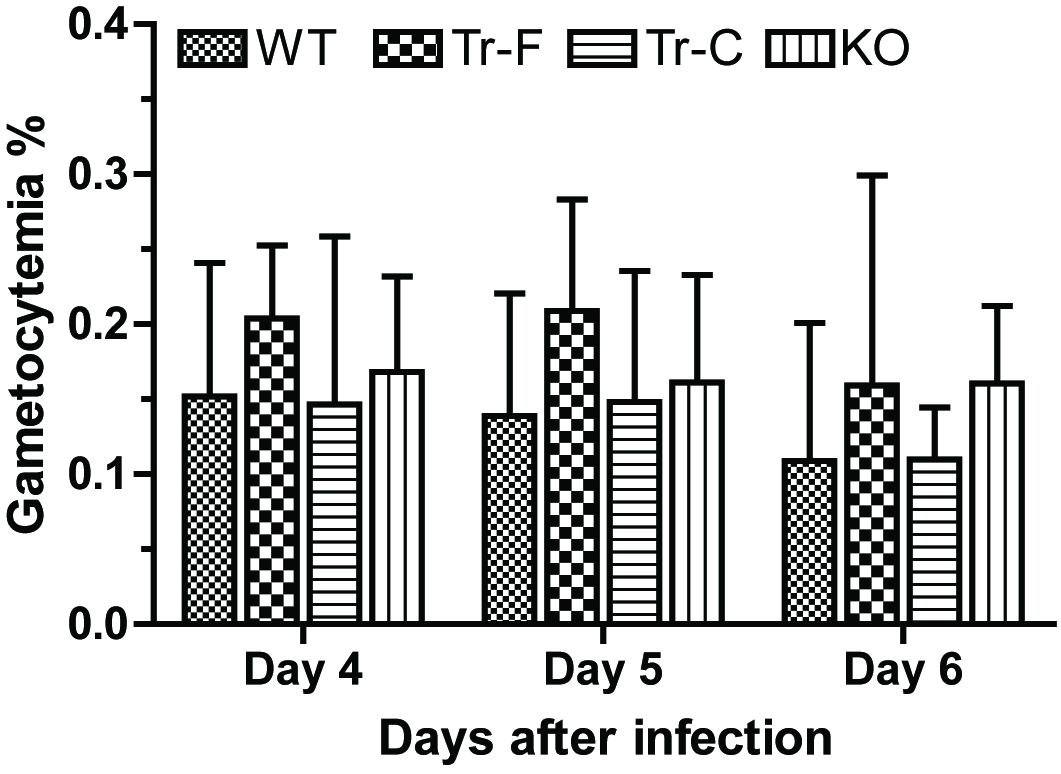

Supplement: FIG S2 [file mbo004184050sf2.tif]

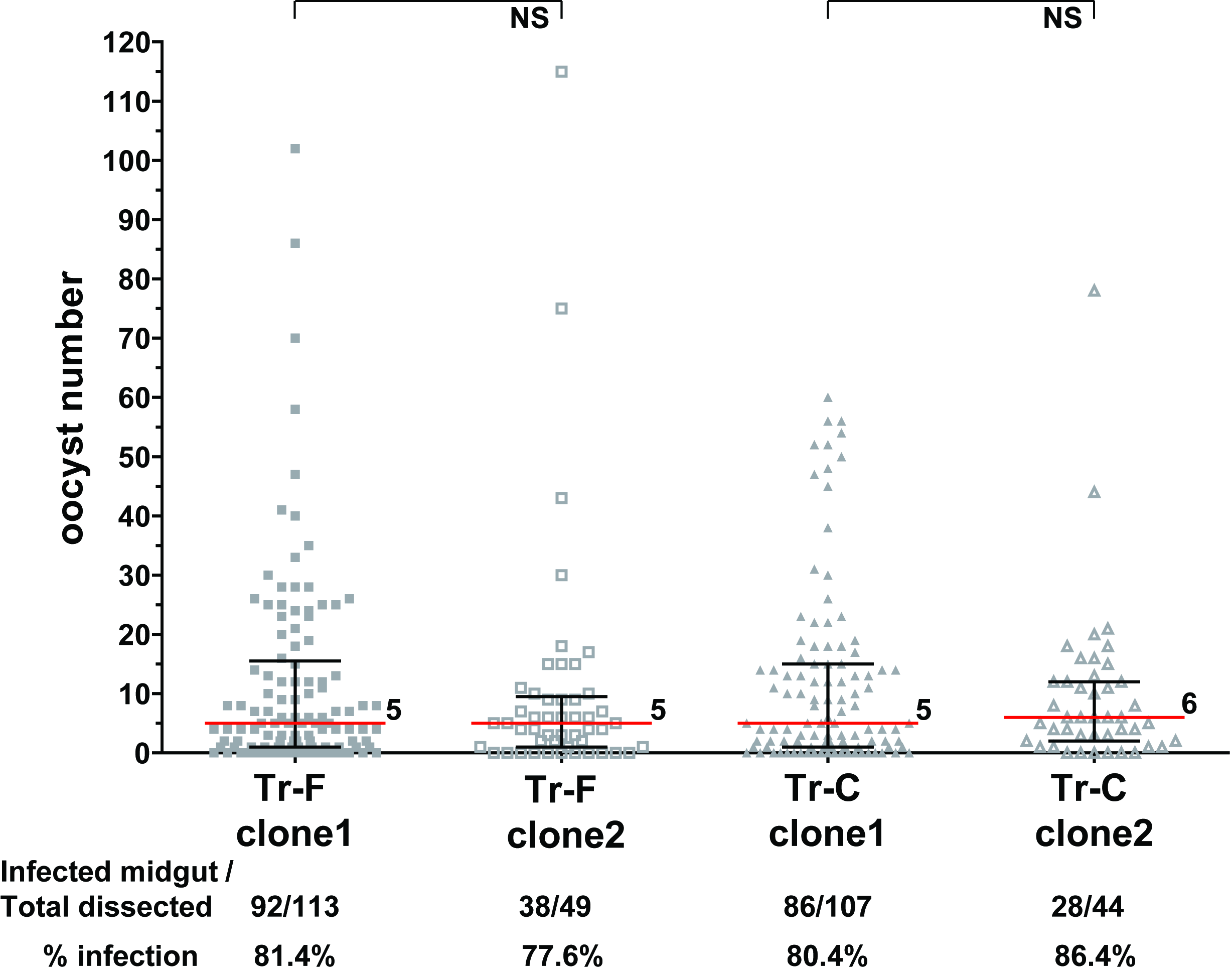

Supplement: FIG S3 [file mbo004184050sf3.tif]
